# Iron and Zinc Homeostasis and Interactions: Does Enteric Zinc Excretion Cross-Talk with Intestinal Iron Absorption?

**DOI:** 10.3390/nu11081885

**Published:** 2019-08-13

**Authors:** Palsa Kondaiah, Puneeta Singh Yaduvanshi, Paul A Sharp, Raghu Pullakhandam

**Affiliations:** 1 Biochemistry Division, National Institute of Nutrition, ICMR, Hyderabad 500 007, India; 2 Department of Nutritional Sciences, Kings College London, London SE1 9NH, UK

**Keywords:** iron, zinc, interactions, DMT1, ZIP4, pancreas, metabolism, homeostasis, intestine, Caco-2 cells

## Abstract

Iron and zinc are essential micronutrients required for growth and health. Deficiencies of these nutrients are highly prevalent among populations, but can be alleviated by supplementation and food fortification. Cross-sectional studies in humans showed positive association of serum zinc levels with hemoglobin and markers of iron status. Dietary restriction of zinc or intestinal specific conditional knock out of ZIP4 (SLC39A4), an intestinal zinc transporter, in experimental animals demonstrated iron deficiency anemia and tissue iron accumulation. Similarly, increased iron accumulation has been observed in cultured cells exposed to zinc deficient media. These results together suggest a potential role of zinc in modulating intestinal iron absorption and mobilization from tissues. Studies in intestinal cell culture models demonstrate that zinc induces iron uptake and transcellular transport via induction of divalent metal iron transporter-1 (DMT1) and ferroportin (FPN1) expression, respectively. It is interesting to note that intestinal cells are exposed to very high levels of zinc through pancreatic secretions, which is a major route of zinc excretion from the body. Therefore, zinc appears to be modulating the iron metabolism possibly via regulating the DMT1 and FPN1 levels. Herein we critically reviewed the available evidence to hypothesize novel mechanism of Zinc-DMT1/FPN1 axis in regulating intestinal iron absorption and tissue iron accumulation to facilitate future research aimed at understanding the yet elusive mechanisms of iron and zinc interactions.

## 1. Introduction

Iron and zinc are essential micronutrients required for growth and sustained health. Physiologically, iron is defined as type 1 nutrient, while zinc is a type 2 nutrient [1]. Type 1 nutrient (i.e., iron, calcium, iodine, vitamins A and B) inadequacy manifests in reductions in stores followed by functional plasma components, whereas in type-2 nutrient (i.e., zinc, protein, sodium and water) deficiencies the clinical symptoms such as impaired growth precedes decline in functional plasma levels. Therefore, assessment of hemoglobin or serum ferritin/transferrin receptor serve as early diagnostic markers of anemia and iron deficiency, while there are no established biomarkers of zinc deficiency [2]. Poor density and bioavailability of iron from typical vegetarian foods is the major etiological factor for the high prevalence of anemia in general population [3,4]. Phytic acid, an abundant secondary metabolite of plant foods, chelates dietary iron, and limits its intestinal absorption. Since phytic acid also inhibits the zinc absorption, higher risk of zinc deficiency is expected in populations with high prevalence of anemia, stunting, and high phytate content of staple diets [2,5]. Given the impact of these deficiencies on general health, particularly in children and pregnant women, correction of these deficiencies through therapeutic or food fortification approaches should be considered.

Iron and zinc combined supplementation trials in humans and animal models have revealed negative interactions, but there are conflicting data on the direction and magnitude of these interactions [6,7]. It has been hypothesized that iron–zinc interactions occur through competition at a specific transport protein during intestinal absorption; however, the exact mechanisms remain elusive. On the other hand, zinc deficiency, investigated via experimental animal models and in vitro studies, gives rise to iron deficiency anemia, tissue and cellular iron accumulation [8,9,10,11]. Cross-sectional studies in humans reveal a positive association of serum zinc levels with hemoglobin and markers of iron status [12,13,14,15]. Studies in intestinal cell culture and experimental animal models have also demonstrated modulation of iron transporter expression and iron regulatory proteins by zinc [11,16,17,18,19]. We have demonstrated that zinc induces iron uptake and transcellular transport in intestinal cells via induction of DMT1 and FPN1 expression [16,17,19,20]. Therefore, zinc appears to be a key modulator of intestinal iron absorption and tissue iron distribution possibly mediated via regulating the DMT1 and FPN1 levels.

Here, we have reviewed the mechanism of iron and zinc homeostasis and their interactions both at the level of intestinal absorption and tissue mobilization in the context of zinc status. From the available evidence, it appears that zinc-DMT1/FPN1 axis is a critical determinant of zinc-deficiency-induced changes in iron homeostasis and helps understand the mechanism of iron and zinc interactions.

## 2. Iron Homeostasis

**Figure 1:**
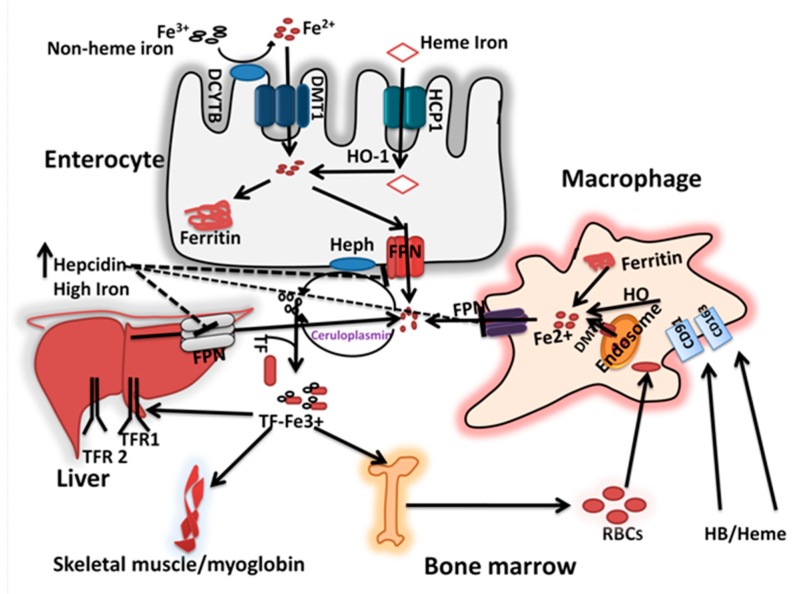
Iron absorption and homeostasis: The dietary non-heme iron is first reduced to ferrous form by Dcytb and is taken up via DMT1 at the apical surface of the enterocytes. Dietary heme iron is taken up via HCP 1 and degraded inside the cell by HO-1 to release iron. Within the enterocyte, the iron is either stored in ferritin or transported to the circulation. At the basolateral membrane, the ferrous iron transported via FPN1, coupled with its oxidation by HEPH. The senescent erythrocytes are phagocytosed by macrophages via CD91/CD163, and are degraded in lysosomal compartments to release iron, which then are excreted into cytosol via DMT1. The iron is then transported out of the macrophage via FPN1 possibly coupled with ceruloplasmin dependent iron oxidation. In the blood, ferric iron is transported bound to Tf, and delivered to the target tissues, the bone marrow, liver and muscle via TfR-dependent mediated endocytosis pathway. When the iron stores are adequate, the hepcidin released from the liver into the blood, which in turn inhibits the FPN1 mediated iron release from intestinal cells and other tissues involved in iron mobilization.

In mammals, iron is highly conserved and there are no obligatory pathways for its excretion. The basal losses of iron through shedding of intestinal cells, sweat, and urine, and increased demand due to infant and adolescent growth spurts and pregnancy is compensated by the concurrent modulation of intestinal iron absorption. In addition, iron is stored and recycled in the body and thus counters short-term dietary inadequacies [21]. The mechanistic aspects of iron absorption, transport, storage, recycling, and their regulation are depicted in Figure 1 and described below.

### 2.1. Iron Absorption and Recycling

Dietary iron exists in either heme (animal foods) or non-heme forms (plant foods), but the latter is the predominant source of iron in the diet [22,23]. Villus epithelial cells, known as enterocytes, in the duodenum and upper jejunum are specialized in rapidly transporting both heme and non-heme iron from the lumen to the blood. The heme iron absorption is mediated by heme carrier protein (HCP1), which undergoes intracellular degradation by heme oxygenase (HO-1) to release iron within the enterocyte [21]. Non-heme iron absorption depends on the solubility of ferric iron in the gastric milieu and its reduction to ferrous form by compounds such as ascorbic acid in the duodenum [21,24]. In addition, soluble ferric iron, possibly bound to peptides, organic acids, or amino acids, is first reduced by Duodenal cytochrome b (Dcytb) [25], before it is taken up by enterocytes via DMT-1, a proton-coupled solute carrier protein [26]. After iron has entered the enterocyte, two pathways for its handling are available: transfer across the basolateral membrane or binding to specific cytoplasmic protein, ferritin. The path taken is governed by the body’s demands for iron. In conditions of iron excess, the metal is oxidized to Fe^3+^ at the ferritin shell before being stored [21,24]. There is evidence that iron stored in ferritin can be re-mobilized by targeting ferritin to autolysosomes in a process called ferritinophagy [27]. When demands are high, iron preferentially passes across the basolateral membrane into portal circulation via sequential action of FPN1 and hephaestin (HEPH) [28,29]. FPN1, a transmembrane protein, abundant at basolateral membrane of polarized enterocytes exports the cellular iron into circulation. Indeed, FPN1 is the only iron exporter to have been identified to date [28,30,31].

Hephaestin, a transmembrane protein with copper-dependent ferroxidase activity, is predominantly present at the basolateral side of polarized enterocytes converts ferrous iron (Fe^2+^) to the ferric (Fe^3+^) form. In the circulation, the apo-transferrin (Tf) binds to the ferric iron and assists in its transport in the blood plasma. Transferrin-bound iron is delivered to the target tissues; most go to the bone marrow, via transferrin receptor (TfR)-mediated endocytosis, and is diverted to functional pools (such as hemoglobin in erythrocytes) or stored in ferritin [32]. Spleen-, liver- and bone marrow-derived macrophages engulf senescent erythrocytes via phagocytosis, and the iron is recycled via FPN1/ceruloplasmin (a copper-dependent ferroxidase) for re-use in metabolism [32,33,34]. It is estimated that about 20 mg/day of iron is required for erythropoiesis, must be met from newly absorbed dietary iron and iron recycled from senescent red blood cells (RBCs) [34]. The typical absorbable dietary iron is in the range of 1.2–2 mg/day, thus recycling of RBC derived iron is critical in maintaining iron balance. In addition to iron status, physiological factors such as inflammation reduce the iron absorption while new tissue growth increases the requirements and thus absorption.

### 2.2. Regulation of Cellular and Systemtic Iron Homeostasis

The cellular iron homeostasis is regulated by iron regulatory protein 1 and 2 (IRP1 and IRP2) by translational control mechanisms. IRPs bind to iron-responsive elements (IRE), stem-loop RNA secondary structures, present in the 3′ and 5′ untranslated regions (UTR) of mRNA transcripts for iron metabolic proteins, including DMT1, ferritin, FPN1, and TfR1 [35]. When intracellular iron concentrations are low, the binding with IRPs is high, which results in the stabilization of transcripts containing IRE in the 3′ UTR (e.g., TfR1 and DMT1), and repression of translation of transcripts containing an IRE in the 5′ UTR (e.g., ferritin and FPN1) (34). The net effect is an increased in TfR1/DMT1 and a decrease in ferritin and FPN1 expression, thus increasing the labile iron concentrations to normal levels. Conversely, when labile iron concentrations are high, the binding of IRPs to IREs is decreased, resulting in reductions in TfR1 and DMT1 and increased ferritin and FPN1 expression, leading to reduced absorption and increased mobilization (34).

Since the sites of iron storage (liver) are different from that of its entry (intestine), these tissues need to cross-talk to regulate iron absorption and mobilization. Hepcidin, a cysteine-rich 25 amino acid cationic peptide synthesized and secreted by the liver, is identified as the key regulator of mammalian iron homeostasis [36,37,38]. Hepcidin, regulated by iron in the liver, inhibits iron absorption by reducing the efflux of iron from storage tissues and from enterocytes. Hepcidin accomplishes this by binding to the iron exporter FPN1, leading to its endocytosis and intracellular degradation, resulting in decreased FPN1-mediated iron transport into extracellular fluids and increased cellular iron retention [38,39]. In addition to iron status, hepcidin expression is also modulated by inflammation, ineffective erythropoiesis, and hypoxia [37].

## 3. Zinc Homeostasis

**Figure 2:**
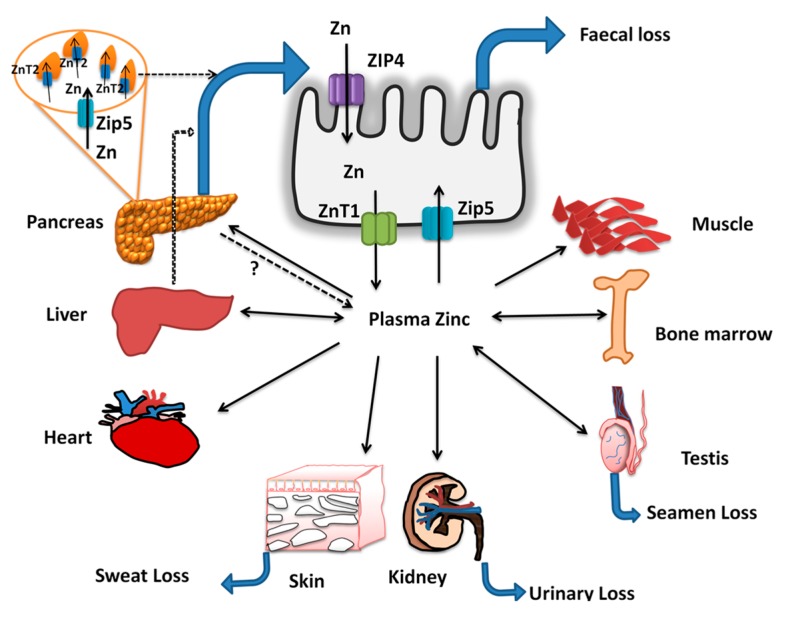
Zinc absorption and homeostasis: Dietary zinc and zinc excreted through pancreatic secretions are absorbed via ZIP4 at the apical surface of the enterocyte and are transported into circulation via ZnT1. The zinc in the plasma bound to albumin (major portion) or in free form is taken up by the peripheral tissues such as liver, bone marrow, testis, kidney, skin, heart, skeletal muscle, and pancreas. In the pancreas, ZIP5 sequesters the zinc from plasma, and it is incorporated in to zymogen granules via ZnT2 and excreted via pancreatic secretions. The absorbed zinc is lost through feces, urine, semen and sweat, among which fecal excretion is sensitive to zinc status of the host. In addition, during zinc insufficiency, the plasma zinc levels are maintained via secretion of zinc only from specific tissues such as liver, bone marrow, and testes while it is strictly conserved in heart, skeletal muscle, skin, and kidney. Thus, enteric excretion of zinc via biliary-pancreatic axis maintains the zinc balance via modulation of excretion during repletion, while zinc depletion is countered via increased ZIP4 mediated absorption in the intestine. Further, during deficiency, few specific tissues release the zinc into the plasma (double arrows) and contribute to the maintenance of plasma zinc pool during inadequate intakes or deficiency.

Homeostatic regulation of zinc metabolism is orchestrated through increased absorption during deficiency and excretion during zinc repletion (Figure 2) [40]. Zinc is ubiquitously present in all tissues, with highest levels found in muscle and bone followed by liver. The whole-body zinc content is stable over a wide range of dietary zinc concentrations indicating efficient homeostatic mechanisms [41]. Zinc absorption and excretion in the gastrointestinal tract are the primary mechanisms for maintaining zinc homeostasis. Zinc absorption takes place throughout the small intestine with the highest rate of absorption occurring in the jejunum, but duodenum contributes to the maximal zinc absorption owing to its exposure to higher zinc concentration after a meal [42]. Thereafter, excess endogenous zinc can be secreted into the intestine and excreted in feces [41]. The balance of intestinal absorption and endogenous losses of zinc through feces are thus two important pathways that regulate the zinc homeostasis. During zinc deficiency or limited dietary zinc intakes, fecal zinc excretion falls with concurrent increase in intestinal absorption, thus conserves the zinc concentration in the tissues/plasma [41,43,44]. On the other hand, during zinc excess, fecal zinc excretion increases while the absorption is not affected. Therefore, an exquisite balance of endogenous losses regulates the whole-body zinc homeostasis, such that the plasma zinc levels remain at steady state except under severe zinc deficiency [41]. The mechanisms involved in intestinal zinc absorption and specific role of pancreas in endogenous zinc excretion are described below.

### Zinc Absorption and Excretion

The identification of two families of zinc transporters, namely ZIP (increases cytosolic zinc) and the ZnT proteins (decreases cytosolic zinc), has contributed considerably to the understanding of both intestinal and systemic zinc homeostasis [45,46,47]. ZIP proteins transport zinc from the extracellular space and intracellular organelles into the cytoplasm, while ZnT proteins function as exporters of intracellular zinc. In humans, 14 members of ZIP and 10 members of ZnT family proteins have been identified and are expressed in tissue-specific manner [48]. Although multiple ZIP family proteins have been identified in the intestine, ZIP4 is the predominant zinc transporter in enterocytes. Genetic mutations in humans or specific knock down in animal models unequivocally indicated the role of ZIP4 in mediating the intestinal zinc absorption [9,48,49]. In addition, up regulation of ZIP4 expression during zinc deficiency and its internalization followed by degradation during zinc supply, indicates that ZIP4 expression is sensitive to cellular zinc levels [44]. ZIP5 is expressed in intestine, pancreas, kidney, and embryonic yolk sac, but is localized predominantly at the basolateral surface of polarized cells and thus is proposed to function in the uptake of zinc from the circulation [50,51]. In contrast to ZIP4, the zinc deficiency induced rapid internalization of ZIP5 in enterocytes [51]. Therefore, the regulation of ZIP4 and ZIP5 controls the flux of zinc across the mucosal cells, thus helps adapting to dynamics of zinc status (Figure 2). Further, higher serosal uptake of zinc via ZIP5 during zinc replete states might increase intestinal zinc pool, leading to degradation of ZIP4 and thus reduced absorption, but this possibility needs to be explored. The zinc absorbed by the enterocytes is either stored as metallothionein or is transported across the basolateral membrane via ZnT1 [52,53]. The abundant expression of ZnT1 at the basolateral surface of the intestine and the severe zinc deficiency due to its mutations infer a specific role for this protein in mediating zinc exit from enterocytes into the circulation [54]. The functional significance of other ZnT and ZIP family members in trafficking cytosolic zinc, and its storage in cell organelles remains an active area of research.

Early studies using radio-tracer methods identified that the exocrine pancreas plays a functional role in zinc excretion. Indeed, pancreas, intestine, and liver have been identified to have very high turnover rates of zinc [55]. In addition, pancreatic acinar cells possess much higher zinc concentrations compared to islet tissue. It is estimated that under normal dietary conditions, 1–2 mg/d Zn enters the digestive tract from the exocrine pancreas [44,56]. Dietary Zn restriction markedly decreases the Zn concentration in both pancreatic tissue and secretions [42,57]. The zymogen granules contain enzymes necessary for digestion and for many, their activity is Zn-dependent [40]. ZnT2 was found to be localized in zymogen granules in acinar cells and its expression is upregulated by zinc via MTF-1 dependent mechanisms [58]. These results imply that ZnT2 is involved in pumping the zinc into zymogen granules and subsequent excretion into gastro-intestinal tract [58]. These observations together suggest that zinc homeostasis is regulated, via modulating the zinc excretion through the entero-pancreatic axis.

## 4. Impact of Zinc Deficiency on Iron Status

Clinical studies in human subjects indicated that serum zinc levels correlate with iron status markers. Hemoglobin, plasma ferritin, mean corpuscular volume (MCV), and red cell distribution width (RDW) were found to be higher in zinc sufficient (>100 µg/dL) compared to zinc deficient (<100 µg/dL) subjects [12]. Similarly, a large cross-sectional study among pregnant women (*n* = 1185) found low serum zinc levels among anemic subjects, and furthermore, serum zinc levels were significantly and positively correlated with hemoglobin [13]. Other studies have shown that the plasma zinc levels were significantly lower among subjects with iron deficiency anemia [12,13]. In addition, low serum zinc reported to be an independent risk factor for anemia among school-age children in New Zealand [14]. These studies together indicate that zinc status is associated with iron metabolism among human subjects. Since iron and zinc inadequacies likely co-present in the diet, the causal role of zinc in influencing iron status cannot be established in these cross-sectional studies. Interestingly, concurrent iron deficiency anemia has been reported among people with *acrodermatitis enteropathica*, a rare genetic disease characterized by zinc deficiency [59], therefore a causal role of underlying zinc deficiency in development of anemia or iron deficiency cannot be excluded.

Studies in experimental animals demonstrated development of iron deficiency anemia and tissue iron accumulation during zinc deficiency. For instance, studies in rats given low zinc diets led to a reduction in iron status parameters such as hemoglobin and RBC number, which could be either due to reduced erythropoiesis or increased catabolism [8,60]. Similarly, dietary zinc restriction of rats led to iron accumulation across multiple tissues, and this is reversed by supplementation with zinc [10]. Furthermore, maternal zinc restriction also resulted in higher tissue accumulation of iron in fetuses, which appears to be stored in the ferritin-hemosiderin fraction [61]. In intestinal specific conditional ZIP4 knockout mice, the intestinal iron and zinc concentrations are significantly reduced at day 4 compared to wild type control while the liver iron remained similar. Interestingly, at day 8, the liver iron concentration in knockout mice was markedly higher despite the fact that liver zinc levels remained unchanged [9]. Adipocyte cell lines grown in zinc deficient medium accumulate iron as a result of increased TfR1 and ferritin and reduced DMT1 levels [11]. Similarly, zinc supplementation has been reported to induce the FPN1 expression in zebra fish gills [62]. These results clearly suggest that zinc status has a profound impact on the intestinal iron absorption and distribution of iron in tissues.

It has also been hypothesized that the zinc might induce iron homeostasis via regulation of hepatic hepcidin expression [63,64]. Hepcidin expression in the liver requires activation of the HJV-BMP-SMAD pathway [64]. Matriptase-2 (TMPRSS6; a hepatocyte plasma membrane associated serine protease) regulates the levels of HJV via its proteolytic degradation. Since matriptase-2 is a zinc-dependent enzyme, it is hypothesized that during the low zinc status, inhibition of matriptase-2 activity might induce hepcidin production. In fact, zinc deficiency is associated with both reduced absorption and increased tissue accumulation of iron; this could be a potential mechanism. However, there is no direct evidence for the association of zinc status (serum zinc) either with matriptase-2 activity or with serum hepcidin levels.

Surprisingly, there are no studies measuring iron absorption in relation to zinc status in humans, possibly due to lack of reliable biomarker of zinc deficiency. However, a study in suckling rat pups demonstrated that during early infancy (at day 10 of parturition) zinc supplementation increases the DMT1 and FPN1 mRNA and protein levels, but these effects are not observed in late infancy (at day 20 of parturition) [18]. This study also demonstrated that zinc supplementation increases the mRNA expression of hepcidin in the liver during early infancy but reduces at late infancy. These results further suggest that zinc modulates the expression of iron metabolic proteins, but these effects could be varied by age and other physiological factors.

## 5. Iron and Zinc Interactions During Absorption

The homeostatic mechanisms controlling iron and zinc metabolism compensate for fluctuations in dietary exposure. However, chronic consumption of foods that have low levels of these dietary metals or abundant concentrations of dietary inhibitors leads to negative balance or deficiency. Considering the high prevalence of anemia and zinc deficiency in populations, supplementing iron and zinc together could be an ideal strategy. Studies in fasting human subjects have indicated a dose-dependent decrease in zinc absorption (25 mg dose) as measured by area under the curves during 4 h time period post-dosing, when supplemented along with non-heme iron, at 2–3-fold molar excess, but not with heme iron [65]. The inhibitory effect of iron on zinc absorption was also found to be higher with ferrous iron than its ferric counterpart. Further, prior administration of therapeutic doses of iron had no impact on zinc absorption [66]. Together, these studies suggest competitive interaction between iron and zinc during intestinal absorption. However, other studies measuring the zinc absorption at more appropriate doses (2.6 mg) by whole-body counting found significant negative interaction of iron on zinc absorption only at 25:1 but not at 2.5:1 ratio [67]. Further, the extent of interaction was either decreased or disappeared when minerals are supplemented with histidine (a zinc chelator) or a test meal, respectively. In agreement, multiple studies reported negative interaction of iron on zinc absorption only when given liquid form (cola or water) but not from meal [6]. Further, consumption of iron fortified foods had no impact on zinc absorption among adult human subjects or infants [68,69]. In addition, a study among pregnant women consuming therapeutic iron doses did not find changes in either serum zinc or exchangeable zinc pools measured using stable isotopes [70]. It is evident from all these observations that negative effect of iron on zinc absorption is only observed when therapeutic doses (2–3-fold excess, or higher, iron relative to zinc) are given to human subjects.

If the interactions of iron and zinc occur at a specific protein site, zinc would also be expected to inhibit the absorption of iron. Indeed, zinc at high doses does reduce the absorption of iron in adult human subjects when fed with liquid, but no such effects were seen when given in a meal [71]. A review of randomized controlled trials of iron and zinc supplementation in human subjects concluded that there is no strong evidence for negative interactions between these minerals [7]. In fact, this review concluded that iron had no impact on zinc status, but zinc appears to have marginal negative impact on iron status, particularly on ferritin levels, a marker of iron stores. In contrast, a study in Peruvian children showed improved hemoglobin, iron status, and reduced diarrhea when iron and zinc are supplemented with a 1 h time gap between zinc and iron doses [72]. Therefore, it is likely that spacing iron and zinc doses augments the response to iron therapy, possibly via reducing the interactions or by increasing the intestinal absorption of iron, as explained later.

A meta-analysis of longer-term zinc supplementation trials indicated no impact of zinc on hemoglobin [73]. Further, zinc supplementation also did not influence the ferritin levels in children [74]. However, this study did indicate that in individuals with severe zinc deficiency or with baseline infections, zinc supplementation is associated with hematological benefits. Similarly, supplementation of iron and zinc together was reported to augment the response to iron supplementation and to reduce the prevalence of diarrhea and to improve motor development and exploratory behavior in children [7,75]. Therefore, the existing evidence suggests that addition of zinc to iron supplementation regimens has no significant negative impact on iron status, and in children at risk of nutritional deficiencies zinc appears to influence the iron status favorably.

## 6. Does Enteric Zinc-DMT1/FPN1 Axis Play a Role in Intestinal Iron Absorption?

It was thought initially that iron and zinc, due to their similar atomic radius and oxidation state, might compete for intestinal absorption at divalent metal ion transporter-1 (DMT1), a proton coupled apical iron transporter in intestinal cells [76]. However, iron but not zinc uptake in intestinal cell models is induced by acidic pH, and neutralizing antibody of DMT1 had no effect on zinc absorption in Caco-2 cells, implying that zinc is not a substrate for DMT1 [77]. However, we have demonstrated that treatment of intestinal cells with zinc increases the iron absorption via induction of mRNA and protein expression of DMT1 [16,19]. The zinc-induced increase in intestinal iron absorption is mediated by IRP2-dependent stabilization of DMT1 mRNA with a concomitant increase in DMT1 protein expression. This pathway requires activation of PI3K signaling [20]. Zinc also increased the expression of FPN1 and basolateral exit of iron in Caco-2 cells. These effects appear to be mediated via metal transcription factor-1 (MTF1) [16,17,78].

We have demonstrated in Caco-2 cells that iron and zinc inhibit the absorption of each other [16,19]. However, pretreatment of Caco-2 cells with zinc led to disappearance of these interactions despite increased DMT1 expression [19]. Based on these results we speculate that iron and zinc interactions at the enterocyte are mediated by non-DMT1 mechanisms. In agreement with this notion, we have shown that treatment of Caco-2 cells with zinc inhibits ZIP14 mRNA expression [17]. Since ZIP14 has been demonstrated to transport both iron and zinc in hepatocytes cells [79], this transporter might mediate the iron and zinc interactions and this possibility needs more systematic verification. In addition, we also demonstrated that zinc deficiency induced by specific chelator in Caco-2 cells also reduces the iron uptake due to delocalization of DMT1 from plasma membrane [17,19]. These results together suggest that intestinal cell zinc status has a profound effect on iron absorption.

As described above, unlike iron, whose homeostasis is regulated by controlling the absorption zinc homeostasis is exclusively regulated by controlling the excretion of endogenous zinc. Although zinc excreted via multiple routes such as urine, sweat, and semen, the enteric secretions constitute a major route of endogenous zinc secretion. Enteric zinc is formed of a combination of dietary zinc and zinc from endogenous secretions derived from bile and pancreatic juice [41,43]. In rats injected with intravenous labeled zinc, significant amounts of zinc appeared rapidly in the gastrointestinal tract, of which about 80% was present in the small intestine. Analysis of tissue uptake of injected zinc showed predominant localization in pancreas and liver [80]. A study in swine indicated that of the total zinc excreted in gastric secretions, pancreatic juice and bile contributes to 60% and 40%, respectively [81]. However, unlike pancreatic zinc, the zinc content of biliary secretions appears to be independent of zinc status [81]. Studies in dogs demonstrated that excretion of zinc though pancreatic secretions is far exceeds that of bile [82]. Further, studies in experimental animals also demonstrated reduction in pancreatic zinc content when fed on zinc deficient diet [57,58,83]. Similarly, studies in human subjects observed that fecal zinc excretion, which in turn is contributed by biliary-pancreatic secretions, is related to the dietary zinc intake and zinc status of host [84]. From these results it is clear that the pancreas has inherent propensity to sequester and excrete zinc, and thus is an important organ in the regulation of zinc homeostasis.

**Figure 3:**
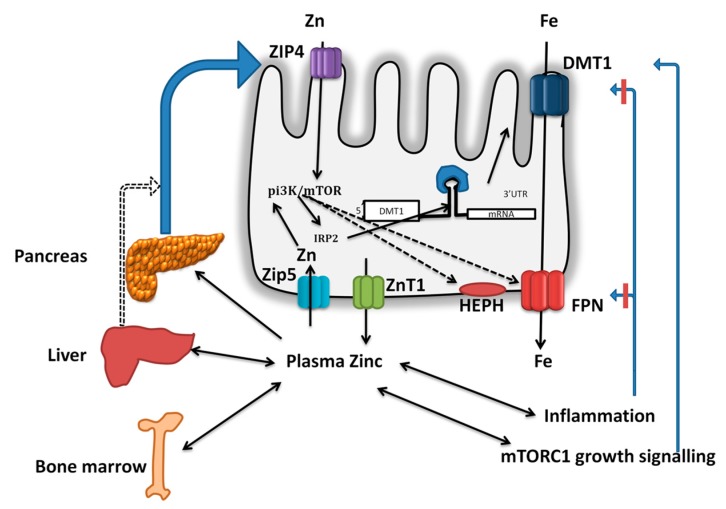
Hypothetical model for direct and indirect effects of zinc on intestinal iron absorption and iron homeostasis: During adequate zinc status and dietary intakes, the biliary-pancreatic zinc secreted into the intestinal lumen stimulates the intestinal iron transport via PI3K-IRP2-DMT1 and FPN1. During inadequate zinc intake, reduced pancreatic zinc levels reduces intestinal iron transporter DMT1 and FPN1 expression, leading to increased retention and inhibition of absorption. Similarly, excretion of zinc from tissues such as liver and bone marrow results in declined tissue zinc, and as a consequence reduced FPN1 expression, leading to reduced secretion for erythropoietic needs. Alternately, zinc might prevent inflammation and thus negate its inhibitory effect on iron absorption. During zinc sufficiency, growth signaling mediated by mTORC1 pathway might increase the iron requirements and thus improve iron absorption.

It is estimated that 2 mg of zinc is lost through pancreatic secretions per day which corresponds to ~12 µmol/L zinc considering the 2.5 L juice produced per day [56,85]. In addition, the dietary intake of zinc is approximately 12 mg/day [86], though the bioavailability of this source of zinc varies significantly depending on other dietary components. The available evidence clearly indicates that enteric zinc excretion responds to the zinc deficiency and zinc repletion to maintain study-state whole-body zinc pool [44,57]. Therefore, we hypothesize that enteric zinc serves as a stimulus for DMT1 and FPN1 expression in intestinal cells and thus increases the intestinal iron absorption (Figure 3). Conversely, during states of zinc deficiency, reduced pancreatic zinc levels and enteric zinc excretion negatively impact intestinal iron absorption. If true, this might account for the observed co-existence of iron deficiency during zinc deficiency described above.

## 7. Physiological Advantages of Zinc Modulating Iron Homeostasis

It is clear from the above observations that zinc status has a marked impact on iron absorption and metabolism. It is possible that this has physiological relevance and metabolic advantage. It is known that iron and zinc compete with various metabolic proteins due to their similar physico-chemical properties. It is established that iron is a pro-oxidant while zinc is seen as an antioxidant [87]. Therefore, excess iron entry into the body needs to be checked to keep the redox status in balance. Regulation of iron absorption and mobilization by zinc might help to ensure appropriate redox balance. On the other hand, zinc is a type 2 nutrient, whose deficiency immediately manifests in reduction in new tissue growth, and adaptation to a lower basal metabolic rate [88]. Zinc has been demonstrated to induce the activity of mTORC1 pathway, a master regulator of growth in mammals [89]. Therefore, direct regulation of iron metabolism by zinc could reflect changing tissue iron requirements depending on growth at different stages of the life course.

## 8. Conclusions

The available evidence from experimental animals, cross-sectional studies in human subjects, and genetic studies clearly point to an association of whole-body zinc status with iron homeostasis. Particularly, underlying zinc deficiency appears to induce iron deficiency by mechanisms that block either intestinal absorption or mobilization of iron from tissues. The in vitro studies in intestinal cell culture models and studies in animals point to a role for zinc in modulating DMT1 and FPN1 expression, respectively. It is therefore possible that compromised zinc status leads to a reduction in pancreatic zinc content, which in turn reduces intestinal iron absorption via a decrease in DMT1 and FPN1 expression. Thus, the net effect of zinc deficiency is mechanistically linked with development of iron deficiency, induced by both reduced intestinal iron absorption and decreased mobilization of iron from storage sites.

Although this review highlights the cross-talk between zinc with intestinal absorption and tissue mobilization of iron, further investigations are warranted to understand the nexus between these important minerals. For example, there is no direct demonstration of the impact of underlying zinc status (i.e., serum zinc) on iron absorption or hepcidin levels in humans. A typical stable isotope-based iron bioavailability study, with short term dietary restriction of zinc, could be a way forward in this direction. In addition, studies are needed to understand the regulation of hepatic iron metabolism by zinc, particularly those regarding the expression/activity of matriptase-2, BMP signaling, and ferritinophagy may shed further light on the impact of zinc on systemic iron metabolism. Since PI3K activation is required for induction of iron absorption (at least in in vitro studies), and dietary ligands such as phytic acid and polyphenols are known inhibitors of this pathway, further studies are needed to understand if the contribution of these inhibitors to the regulation of iron absorption extends beyond chelation of these metals. In addition, studies are also needed to understand both short- and long-term consequences of dietary zinc restriction on iron metabolism, particularly the mRNA and protein expression of iron metabolic proteins and the effects on distribution and concentrations of metals between different tissues.

It is generally accepted that iron and zinc deficiencies coexist, and it is clear from the above evidence that they do interact negatively when supplemented together. Since baseline zinc deficiency also adversely affects the iron absorption, correction of zinc status might improve the outcomes of iron therapy in populations at risk of zinc deficiency. Furthermore, since zinc rapidly induces the intestinal iron absorption (4 h), alternate supplementation of zinc followed by iron with a time gap is also expected to improve the response to iron therapy, and indeed such evidence already exists [72].
